# Vaccine antigen-based genotyping of *Bordetella pertussis* by direct Sanger sequencing of clinical samples in Peru from 2018 to 2019

**DOI:** 10.1128/spectrum.02004-24

**Published:** 2025-05-14

**Authors:** Eduardo Juscamayta-López, Betsabé Vega-Abad, Faviola Valdivia, María Pía Soto, Helen Horna, Ruth García-de-la-Guarda

**Affiliations:** 1 Centro Nacional de Salud Pública, Instituto Nacional de Salud 54719, Lima, Peru; 2 Facultad de Ciencias Biológicas, Universidad Nacional Mayor de San Marcos 569249 , Lima, Peru; Laboratory Corporation of America Holdings, Burlington, North Carolina, USA

**Keywords:** *Bordetella pertussis*, pertussis, sequencing, vaccine antigen gene, allelic variant, genotype, Peru

## Abstract

Despite widespread vaccination, pertussis (caused by *Bordetella pertussis*) persists in many countries, frequently causing outbreaks and severe cases in infants. The resurgence of pertussis may be due to genetic changes in the vaccine antigens of circulating *B. pertussis* strains. However, current typing methods, which depend on bacterial cultures, hinder our understanding of *B. pertussis* genotypes, especially in developing countries. This study aimed to analyze vaccine antigen-based genotypic variants (*ptxP*, *ptxA*, *fim3*, and *prn*) of *B. pertussis* in Peru from 2018 to 2019 via direct Sanger sequencing of nasopharyngeal swabs (*n* = 96). PCR-based sequencing was successful for the genes *ptxP* in 86% (83/96), *ptxA* in 100% (96/96), *fim3* in 75% (72/96), and *prn* in 68% (65/96) of the samples. The *ptxP3* variant was found in 100% (83/83), *ptxA1* in 100% (96/96), *fim3-1* in 97.3% (70/72), *fim3-2* in 2.7% (2/72), and *prn2* in 100% (65/65) of the samples. Sixty-three samples yielded a complete allelic profile, with genotype VI (*ptxP3-ptxA1-fim3-1-prn2*) predominating nationwide (96.8%), mainly in Lima (29.5%), Amazonas (13.1%), Callao (11.5%), and La Libertad (11.5%). Genotype VII (*ptxP3-ptxA1-fim3-2-prn2*) was less common (3.2%), found in Lima (50%) and Callao (50%). The predominance and expansion of genotype VI suggested the presence of biological traits linked to infection, possibly due to the *ptxP3* allele, such as high respiratory colonization or increased pertussis toxin production, which could potentially increase disease transmission and severity. These findings will facilitate Peru’s ability to monitor and control *B. pertussis*, improving public health responses and reducing the outbreak incidence and severity.

**IMPORTANCE:**

Despite widespread vaccination, pertussis (caused by *Bordetella pertussis*) still causes severe outbreaks in infants worldwide. Genetic changes in the vaccine antigens of *B. pertussis* strains may drive this resurgence. Current culture-based typing methods limit our understanding of these genotypes, particularly in developing countries. This study provides valuable insights into the genotypic variability of *B. pertussis* in Peru from 2018 to 2019, employing an isolation-free genotyping method allowing the direct Sanger sequencing of vaccine antigen genes from clinical samples. These findings can enhance public health decision-making by improving our understanding of the genetic changes that drive severe pertussis outbreaks, particularly in developing countries that use whole-cell vaccines. This knowledge enables rapid outbreak responses, improved vaccine strategies, and strengthened surveillance, prevention, and control measures.

## INTRODUCTION

Pertussis, caused by a gram-negative coccobacillus named *Bordetella pertussis*, is a highly contagious respiratory disease, responsible for an estimated 24.1 million cases and approximately 160,700 deaths annually worldwide (1). Despite being preventable by vaccination, pertussis has not been fully eradicated and remains endemic in many countries, causing outbreaks and greater severity in infants under 6 months of age, particularly those who are either not immunized or partially immunized (2). Thus, pertussis poses a significant public health concern globally. Currently, immunization against pertussis involves both whole-cell vaccines (WCVs) and acellular vaccines (ACVs) (3). While WCVs are based on killed *B. pertussis*, ACVs contain antigens such as pertussis toxin, pertactin and filamentous hemagglutinin and, in some formulations, type 2 and type 3 fimbriae (4). Pertussis resurgence may have been triggered by genotypic variations of these vaccine antigens in circulating *B. pertussis* strains, potentially giving rise to new variants of the microorganism that are not effectively targeted by current vaccines. Studies have shown the selective pressure exerted by WCVs and ACVs on *B. pertussis* populations (3, 5). For example, *B. pertussis* strains that harbor the *ptxP3* allelic variant have been found to be prevalent in developed countries using ACVs (6).

In Peru, pertussis is controlled by the administration of the pentavalent DTwP-HepB-Hib vaccine (diphtheria, tetanus toxoids, WCV, hepatitis B, and *Haemophilus influenzae* type b), which is administered in three doses at ages 2, 4, and 6 months, followed by boosters with DTwP at 18 months and 4 years (7). Despite this health strategy, *B. pertussis* continues to cause pertussis infections, mainly in nonvaccinated infants younger than 3 months of age (8). Pertussis remains endemic, with periodic outbreaks reported across various regions (9, 10). In 2018 and 2019, 578 and 502 cases were reported, respectively. Tragically, 11 deaths occurred in 2018, all in infants under 1 year, and 12 deaths in 2019, including 9 in infants under 1 year and 3 in children aged 1–4 years (11). Despite the ongoing burden of pertussis, there is limited knowledge about the circulating *B. pertussis* genotypes in Peru, making it unclear whether new variants have emerged. Regular monitoring of the pathogen and updating the genotypic profile of circulating strains are vital for effective public health strategies. However, traditional typing methods face significant limitations due to their reliance on bacterial culture, which is hindered by low sensitivity and the requirement for viable organisms, posing challenges for widespread surveillance.

The aim of this study was to analyze vaccine antigen-based genotypic variants of *B. pertussis* in Peru from 2018 to 2019 using an alternative molecular typing method that is independent of culture and is based on direct Sanger sequencing of clinical specimens. This research approach was adopted to provide a comprehensive overview of the most prevalent *B. pertussis* genotypes circulating in the affected regions of Peru. The resulting data will supply evidence to strengthen surveillance efforts and guide the development of preventive or therapeutic interventions, particularly those aimed at protecting vulnerable populations such as infants. Additionally, this information will allow the evaluation of the impact of WCVs on the *B. pertussis* population and provide evidence for decision-making in response to pertussis outbreaks and for the prevention and control of the disease.

## MATERIALS AND METHODS

### Clinical samples

A retrospective cross-sectional study involving all DNA samples from nasopharyngeal swabs obtained from patients with suspected pertussis across the nation was conducted. These samples were submitted to the Laboratory of Special Bacteriology (BAES) at the National Institute of Health (INS) between 2018 and 2019 for diagnostic confirmation via multitarget quantitative polymerase chain reaction (multitarget qPCR). The analysis included two assays: a multiplex assay targeting the insertion sequences IS*481*, pIS*1001*, and hIS*1001*, with human *RNaseP* as an internal control, and a singleplex assay targeting the pertussis toxin S1 subunit (*ptx*S1) (12, 13). Nucleic acid samples from clinical samples preserved at −80°C at BAES-INS were randomly selected based on the following inclusion criteria: (i) DNA samples from nasopharyngeal swabs obtained from patients with suspected pertussis nationwide, submitted to INS for diagnostic confirmation between 2018 and 2019, and (ii) a positive result for *B. pertussis* DNA by multitarget qPCR. Samples were excluded if they had (i) insufficient volume (<50 µL) for molecular analysis or (ii) an indeterminate result for pertussis diagnosis by multitarget qPCR. The selected samples (*n* = 198) were analyzed via real-time PCR targeting the *ptxS1* gene, which encodes the pertussis toxin (Table 1), following the protocol described by Juscamayta-López et al. (13) and Tatti et al. (12). Only positive DNA samples with a cycle threshold (CT) value of less than 30 were used to ensure the highest bacterial load of the pathogen in the sample, as recommended in previous studies (14, 15).

**TABLE 1:** Real-time PCR of the *ptx*S1 gene^*a*^

Target	Primer/probe	5′ f	Sequence	3′ q	C (µM)
*ptxP-F*	402U16_F		CGCCAGCTCGTACTTC		0.7
*ptxP-R*	442L15_R		GATACGGCCGGCATT		0.7
*ptxA-F*	419U22P_P	Texas Red	AATACGTCGACACTTATGGCGA	BHQ2	0.3

^*a*^ f, fluorophore; q, quencher; C, concentration. Empty cells refer to the forward and reverse primers, which are not labeled with fluorophores. Only the probe is conjugated with a fluorophore.

### Culture and DNA extraction

The reference *B. pertussis* strain Tohama I ATCC-BAA-589 was reactivated on Bordet-Gengou agar plates supplemented with 15% sheep blood and incubated at 37°C for 48–96 h (16) to serve as controls in the PCR and sequencing assays. Bacterial DNA was extracted using the PureLink Genomic DNA Mini Kit (Invitrogen, Waltham, MA, USA) according to the manufacturer’s protocols. Overall, bacterial cells were lysed in 200 µL of lysis solution, and the DNA was eluted in 100 µL of elution buffer. The extracted DNA was then examined via multitarget qPCR. The concentration and quality of the DNA were assessed using a spectrophotometer (Denovix, USA). Finally, the genomic DNA was stored at −20°C until further use.

### Touchdown PCR optimization

A touchdown PCR assay was individually optimized to increase the specificity and sensitivity for direct detection of genes encoding vaccine antigens or virulence factors in nasopharyngeal swab samples. These genes include the pertussis toxin promoter (*ptxP*), pertussis toxin subunit 1 (*ptxA*), and fimbriae 3 (*fim3*). The pertactin gene (*prn*) was amplified via conventional PCR. The primers used for amplification are detailed in Table 2.

**TABLE 2:** Primers used in the touchdown PCR assays for detecting virulence genes

Primer	Sequence (5′−3′)	Gene	Size (bp)	Reference
*ptxP-F*	AATCGTCCTGCTCAACCGCC	*ptxP*	1,000	(17)
*ptxP-R*	GGTATACGGTGGCGGGAGGA			
*ptxA-F*	CCCCTGCCATGGTGTGATC	*ptxA*	1,200	(17)
*ptxA-F*	TCAATTACCGGAGTTGGGCG			
*fim3-F*	GACCTGATATTCTGATGCCG	*fim3*	1,000	(18)
*fim3-R*	CGCAAGGCTTGCCGGTTTTTTTTGG			
*prn-F*	GCCAATGTCACGGTCCAA	*prn^a^*	600	(19)
*prn-R*	GCAAGGTGATCGACAGGG			

^*a*^ This gene was amplified via conventional PCR.

Amplifications were performed in a Biometra Tone thermocycler (Analytik Jena, Germany). Each reaction mixture had a total volume of 25 µL, and the conditions were optimized as follows: for the *ptxP* gene, the reaction mixture consisted of 1× PCR buffer, 0.2 mM dNTPs, 1.5 mM MgCl_2_, 1 µM primers *ptxP-F* and *ptxP-R* (17), 1.25 U/µL Taq DNA polymerase (Thermo Fisher Scientific, USA), and 5 µL of DNA template. The cycling conditions were 95°C for 3 min, followed by 10 cycles of 95°C for 30 s, 68–59°C for 45 s, and 72°C for 75 s; 35 cycles of 95°C for 30 s, 62°C for 45 s, and 72°C for 75 s; and a final extension at 72°C for 5 min. For the *ptxA* gene, the reaction mixture consisted of 1× PCR buffer, 0.2 mM dNTPs, 1.5 mM MgCl_2_, 0.2 µM primers *ptxA-F* and *ptxA-R* (17), 1.25 U/µL Taq DNA polymerase (Thermo Fisher Scientific, USA), and 5 µL of DNA template. The cycling conditions were 95°C for 3 min, followed by 10 cycles of 95°C for 30 s, 72–63°C for 45 s, and 72°C for 1 min; 25 cycles of 95°C for 30 s, 63°C for 45 s, and 72°C for 75 s; and a final extension at 72°C for 5 min. For the *fim3* gene, the reaction mixture consisted of 1× PCR buffer, 0.2 mM dNTPs, 1.5 mM MgCl_2_, 0.3 µM primers *fim3-F* and *fim3-R* (18), 1.25 U/µL Taq DNA polymerase (Thermo Fisher Scientific, USA), and 5 µL of DNA template. The cycling conditions were 95°C for 3 min, followed by 10 cycles of 95°C for 30 s, 67–58°C for 45 s, and 72°C for 1 min; 35 cycles of 95°C for 30 s, 56°C for 45 s, and 72°C for 75 s; and a final extension at 72°C for 5 min. For the *prn* gene, the reaction mixture consisted of 1× PCR buffer, 0.2 mM dNTPs, 1.5 mM MgCl_2_, 0.3 µM primers *prn-F* and *prn-R* (19), 1.25 U/µL Taq DNA polymerase (Thermo Fisher Scientific, USA), and 5 µL of DNA template. The cycling conditions were 95°C for 3 min, followed by 30 cycles of 95°C for 30 s, 52°C for 45 s, and 72°C for 1 min, with a final extension at 72°C for 5 min.

The PCR products were visualized via 2% agarose gel electrophoresis at 110 V for 60 min. Prior to electrophoresis, the products were mixed (5:3) with 6× DNA Tritrack (Thermo Fisher Scientific, USA). The average size of the obtained amplicons was determined using the GeneRuler 100 bp Plus DNA Ladder (Thermo Fisher Scientific, USA).

### Direct Sanger sequencing of virulence genes from clinical samples

Virulence-associated genes (*ptxP*, *ptxA*, *fim3*, and *prn*) were amplified directly from DNA extracted from nasopharyngeal swabs (*n* = 96) via the optimized conditions described above. The amplified products were purified directly from the final PCR, except for the *prn*-PCR products, which were purified from the agarose gel using a PCR Clean-Up Kit (Geneaid, Taiwan). Both strands of the purified products were sequenced using the primers listed in Table 2. Sequencing reactions were carried out with the BigDye Terminator v3.1 Cycle Sequencing Kit (Applied Biosystems, USA), and the resulting products were analyzed via the Applied Biosystems 3500 Genetic Analyzer (Applied Biosystems, USA).

### Sequence analysis

Sequence analyses were performed using Geneious Prime software (http://www.geneious.com). Sequencing chromatograms were evaluated for quality and trimmed at the 5′ and 3′ ends to remove low-quality sequences with an error probability threshold of 5%. The forward and reverse sequences of each gene (*ptxP*, *ptxA*, *fim3*, and *prn*) were assembled and aligned using the *B. pertussis* strain Tohama I (GenBank accession number: NZ_CP039022) as a reference to identify mutations. Allelic variants were determined by comparing detected mutations with known single nucleotide polymorphisms (SNPs) associated with the *ptxP*, *ptxA*, *fim3*, and *prn* genes. Genotypes were assigned according to the nomenclature proposed by van Gent et al*.* (20).

### Statistical analysis

Statistical analysis was conducted using Stata/MP v16.1, with statistical significance set at *P* < 0.05. Fisher’s exact test was used to compare proportions between categorical variables.

## RESULTS

### Direct PCR-based sequencing of clinical samples

**Fig 1:**
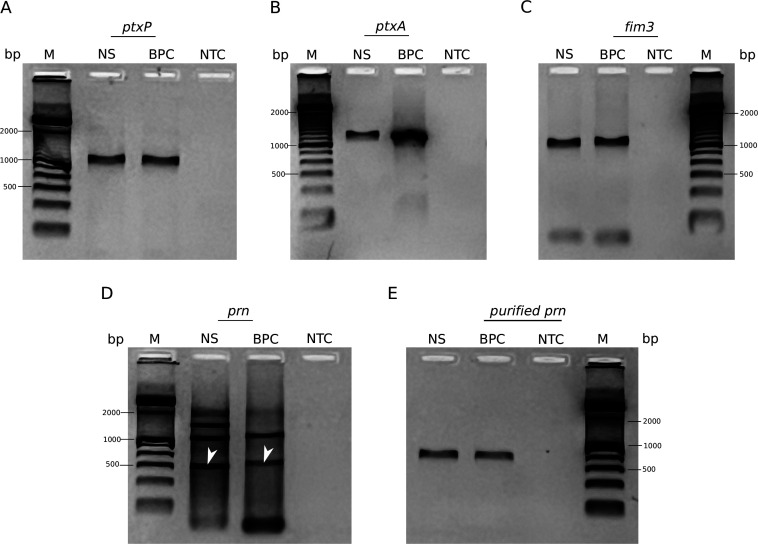
Agarose gel electrophoresis of PCR products from vaccine antigen-based genes detected in clinical samples. Specific products obtained from touchdown PCR of the (**A**) *ptxP*, (**B**) *ptxA*, and (**C**) *fim3* genes. Amplicons obtained from (**D**) *prn*-PCR (arrow) and (**E**) specifically purified via agarose gel electrophoresis. BPC, *B. pertussis*-positive control; M, 100 bp DNA ladder; NS, nasopharyngeal swab sample positive for *B. pertussis* DNA; NTC, no-template control.

The optimized touchdown PCR assay successfully amplified the virulence genes *ptxP*, *ptxA*, and fim3 (Fig. 1A through C), while the specific amplicon of the *prn* gene was purified from agarose gel (Fig. 1D and E). PCR-based sequencing of these genes was performed on 96 nasopharyngeal swab samples collected nationwide from individuals suspected of having pertussis between 2018 and 2019. These samples tested positive for *B. pertussis* DNA using multitarget qPCR conducted at INS-Peru. The singleplex assay targeting *ptx*S1 produced values ranging from 17.77 to 29.91.

### Allelic variants and genotypes of *B. pertussis* circulating in Peru

**Fig 2:**
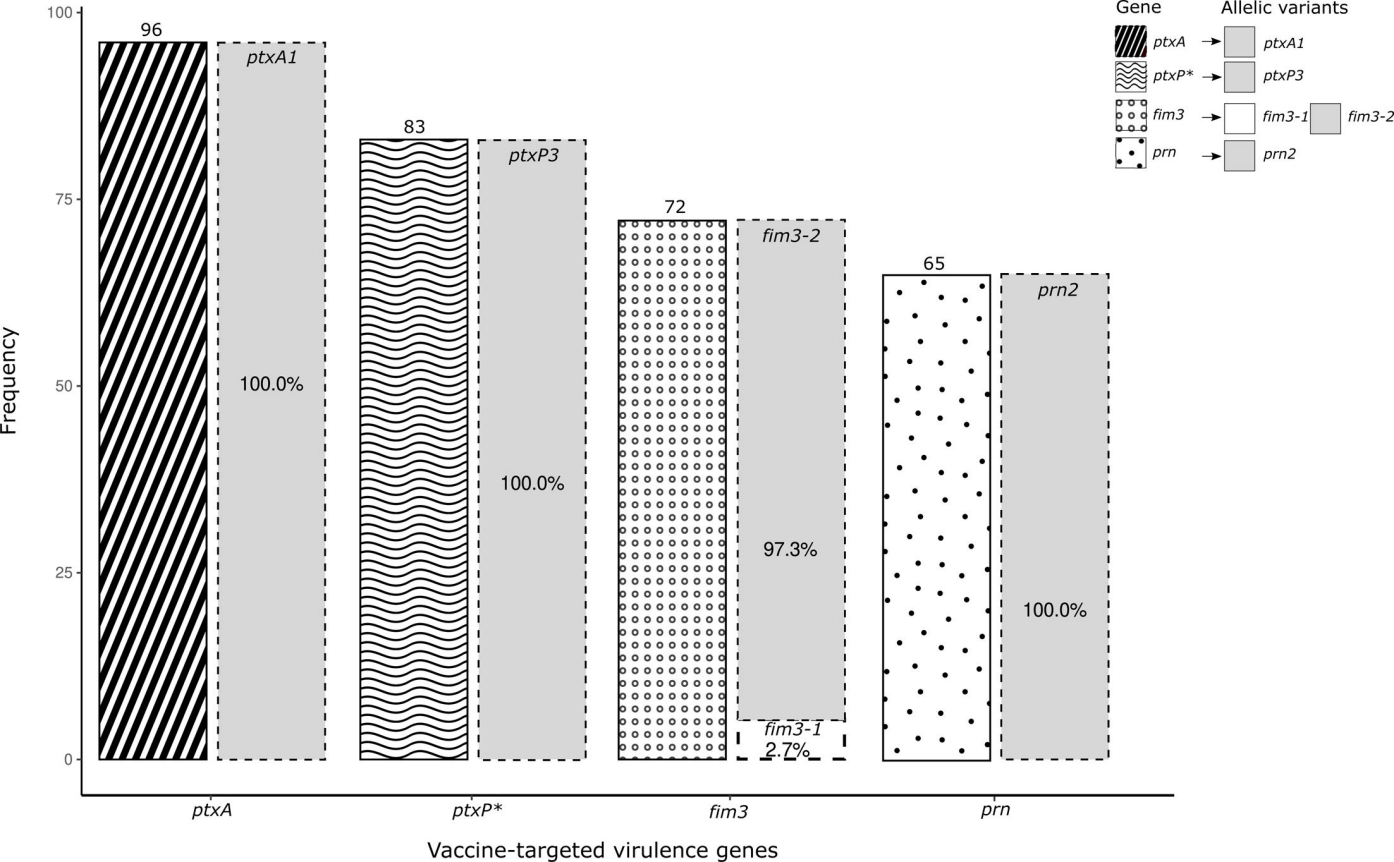
Frequency of vaccine-targeted virulence genes and allelic variants of *Bordetella pertussis* detected in nasopharyngeal swab samples (*n* = 96). Allelic variants are shown as a percentage of the total number of genes detected in clinical samples. Genotyping based on these loci has been extensively studied and is well-documented markers for tracking genotypic variations relevant to vaccine antigens and epidemiological shifts (21). *ptxP refers to the promoter region of the pertussis toxin gene.

Among the 96 samples analyzed, PCR-based sequencing results were obtained for the genes *ptxP* in 86% (83/96), for *ptxA* in 100% (96/96), for *fim3* in 75% (72/96), and for *prn* in 68% (65/96) of the samples (Fig. 2). The allelic variants of *B. pertussis* identified were *ptxP3* (position 61 bp: TGG to TAG) in 100% (83/83), *ptxA1* (position 730 bp: TAG to TGG) in 100% (96/96), *fim3-1* in 97.3% (wild type, 70/72), and *fim3-2* (position 321 bp: GCG to GAG) in 2.7% (2/72) of the sequenced samples, and 100% (65/65) of the *prn*-positive samples harbored the mutation pattern according to *prn2* (5) (Fig. 2).

As shown in Table 3, among the samples carrying the *ptxP3* variant, 33% (27/83) were from the Lima region, 11% (9/83) were from Callao, 11% (9/83) were from La Libertad, and 10% (8/83) were from Amazonas. For the *ptxA1* variant, 29% (28/96) were observed in Lima, 14% (13/96) were in La Libertad, and 11% (9/96) were in Callao. The *fim3-1* allelic variant was found in 26% (18/70) of the samples from Lima, 13% (9/70) were from La Libertad, 13% (9/70) were from Callao, and 11% (8/70) were from Amazonas. Conversely, the *fim3-2* allele was found in 50% (1/2) of the samples from both Lima and Callao. Finally, 31% (20/65) of the prn2 variant samples were from Lima, 12% (8/65) were from Callao, 12% (8/65) were from La Libertad, and 12% (8/65) were from Amazonas.

**TABLE 3:** Distribution of identified *Bordetella pertussis* allelic variants by region in Peru from 2018−2019

Region of Peru	Identified *B. pertussis* allelic variants				
	*ptxP3*	*ptxA1*	*fim3-1*	*fim3-2*	*prn2*
Lima	27	28	18	1	20
Callao	9	11	9	1	8
La Libertad	9	13	9	0	8
Amazonas	8	8	8	0	8
Puno	6	8	6	0	5
Loreto	5	5	4	0	4
Ancash	5	5	3	0	3
Cajamarca	4	5	3	0	2
San Martín	3	3	2	0	2
Lambayeque	2	2	2	0	2
Cusco	1	3	2	0	0
Arequipa	1	2	2	0	1
Ayacucho	1	1	1	0	1
Ucayali	1	1	1	0	1
Huancavelica	1	1	0	0	0
Total	83	96	70	2	65

**Fig 3:**
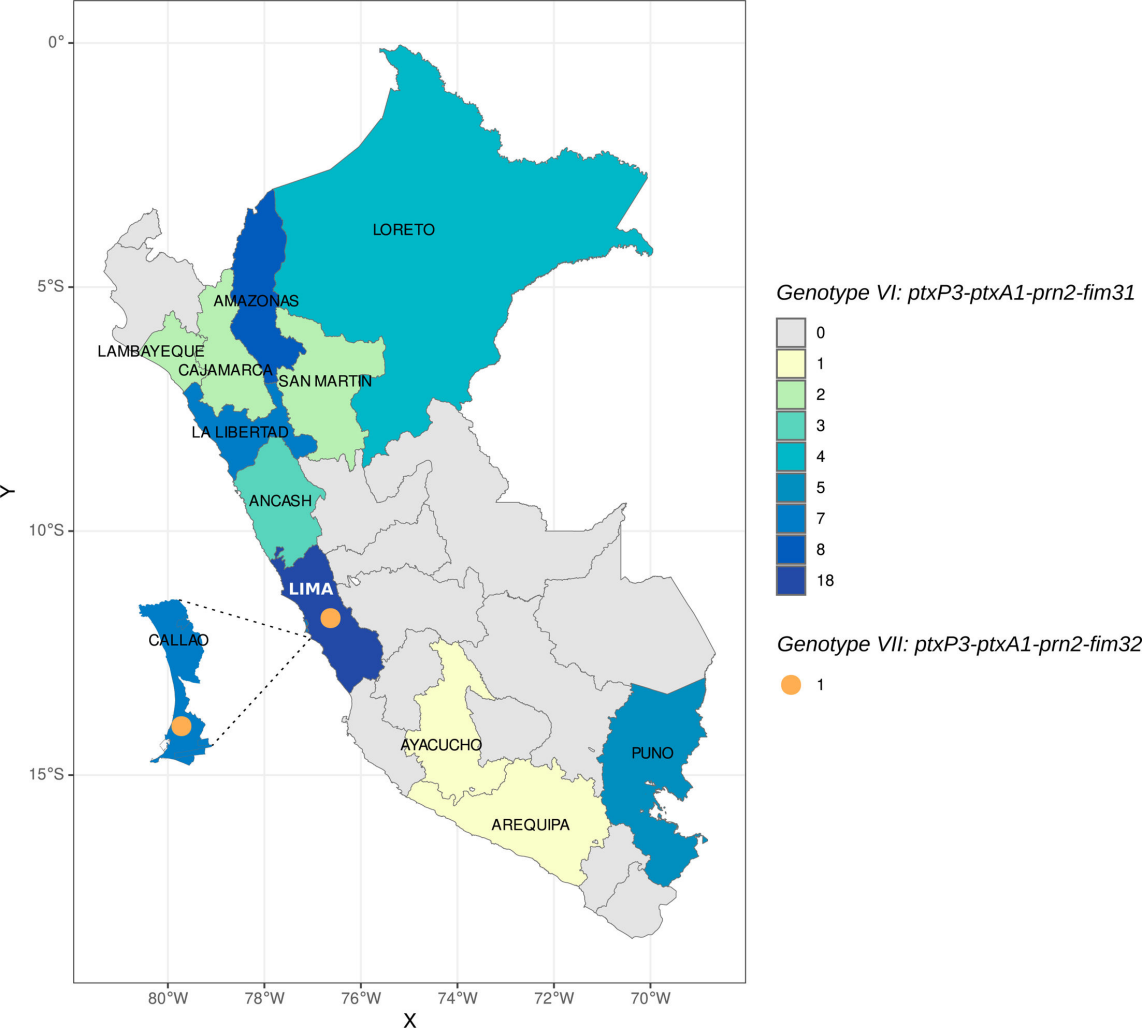
Distribution of *B. pertussis* genotypes circulating in various regions of Peru from 2018 to 2019. The map was created using R (https://www.r-project.org/).

Among a total of 96 samples, 63 presented a complete allelic profile for genotype assignment according to the nomenclature of van Gent et al. Two genotypes were identified. Genotype VI (*ptxP3-ptxA1-fim3-1-prn2*) predominated nationwide (96.8%), with higher frequencies observed in Lima (29.5%), Amazonas (13.1%), Callao (11.5%), and La Libertad (11.5%). Genotype VII (*ptxP3-ptxA1-fim3-2-prn2*) was less common (3.2%) and was found in Lima (50%) and Callao (50%) (Fig. 3; Table 4).

**TABLE 4:** Frequency of *Bordetella pertussis* genotypes identified in Peru from 2018 to 2019

Genotype	Allelic profile	Frequency	Percentage	Cumulative percentage
VI	*ptxP3-ptxA1-fim3-1-prn2*	61	96.8%	96.8%
VII	*ptxP3-ptxA1-fim3-2-prn2*	2	3.2%	100.0%
Total		63	100.0%	100.0%

Among the total number of *B. pertussis* genotypes identified (*n* = 63), 54% were from female patients. However, there were no statistically significant differences between genotypes VI (predominant) and VII among females (*P* = 1.000; 54.1% vs 50.0%, respectively) (Table 5). The age distribution did not significantly differ between genotypes (*P* = 0.444), with genotype VI predominant in infants under 2 months of age (30 cases) compared with genotype VII (1 case). The distribution according to geographic region did not significantly differ between genotypes VII and VI (*P* = 0.912). Additionally, hospitalization rates were similar between genotypes VI (76.5%) and VII (100%), with no statistically significant difference (*P* = 1.000) (Table 5).

**TABLE 5:** Bivariate analysis comparing the clinical and epidemiological characteristics of pertussis cases among circulating genotypes in Peru from 2018 to 2019 (*n* = 63)

Characteristics	Genotype VI (*n* = 61) *n* (%)	Genotype VII (*n* = 2) *n* (%)	*P* value^a^
Sex			1.000
Female	33 (54.1)	1 (50.0)	
Male	28 (45.9)	1 (50.0)	
Age (months)			0.444
1a < 2m	30 (49.2)	1 (50.0)	
2a < 4m	6 (9.8)	1 (50.0)	
4a < 6m	5 (8.2)	0 (0.0)	
>6m	20 (32.8)	0 (0.0)	
Geographic region			0.912
Ancash	3 (4.9)	0 (0.0)	
Amazonas	8 (13.1)	0 (0.0)	
Arequipa	1 (1.6)	0 (0.0)	
Ayacucho	1 (1.6)	0 (0.0)	
Cajamarca	2 (3.3)	0 (0.0)	
Callao	7 (11.5)	1 (50.0)	
La Libertad	7 (11.5)	0 (0.0)	
Lambayeque	2 (3.3)	0 (0.0)	
Lima	18 (29.5)	1 (50.0)	
Loreto	4 (6.6)	0 (0.0)	
Puno	5 (8.2)	0 (0.0)	
San Martin	2 (3.3)	0 (0.0)	
Ucayali	1 (1.6)	0 (0.0)	
Clinical symptoms			
Paroxysmal cough			0.111
No	2 (3.9)	1 (50.0)	
Yes	49 (96.1)	1 (50.0)	
Noisy inspiration at the end of a coughing episode			0.492
No	25 (49.0)	0 (0.0)	
Yes	26 (51.0)	2 (100.0)	
Vomiting after coughing			1.000
No	18 (35.3)	1 (50.0)	
Yes	33 (64.7)	1 (50.0)	
Pneumonia			1.000
No	34 (66.7)	2 (100.0)	
Yes	17 (33.3)	0 (0.0)	
Hospitalization			1.000
No	12 (23.5)	0 (0.0)	
Yes	39 (76.5)	2 (100.0)	

^a^ Calculated by Fisher’s exact test.

## DISCUSSION

*B. pertussis* is a challenging microorganism to isolate because various factors affect its viability, including the timely collection and transportation of samples, specialized culture requirements, and whether the patient was vaccinated or received antibiotics against pertussis (22). Current typing methods rely on pathogen isolation, which has restricted understanding of genotypic diversity in our country. In this study, we detected genotypic variants of *B. pertussis* circulating in Peru between 2018 and 2019 using an alternative isolation-free typing method based on the sequencing of vaccine antigen genes (*ptxP*, *ptxA*, *fim3*, and *prn*) directly from nasopharyngeal swab samples.

We detected the allelic variants *ptxA1* and *ptxP3* in all the clinical samples analyzed for the *ptxA* (96/96) and *ptxP* (83/83) genes, respectively (Fig. 2). Among the 11 allelic variants of *ptxP* (*ptxP1* to *ptxP11*), *ptxP3* is prevalent worldwide (17, 23). These two allelic variants (*ptxA1* and *ptxP3*) have been reported in the United States (24), Mexico (25), Argentina (26), Australia (27), China (28), Japan (29), South Africa (30), and various European countries, including Italy, France, Spain, and Belgium (5). Notably, *B. pertussis* strains carrying the *ptxP3* allelic variant have been associated with increased pertussis toxin production, increased transmission, and enhanced virulence (17, 31). Experimental evidence further indicates that *ptxP3* strains possess a competitive advantage over other variants (32), which could explain their widespread presence in Peruvian regions with high pertussis prevalence, such as Lima, Callao, La Libertad, and Amazonas (9, 10) (Table 3).

The *prn* gene exhibits globally circulating allelic variants, including *prn1*, *prn2*, and *prn3*. Historically, *prn1* predominated until 1996, when the *prn2* allele began to emerge, coinciding with the introduction of the ACV (33). Although *prn2* circulation has been associated with regions where the ACV is administered, our study identified this variant in 100% of the analyzed samples (Fig. 2), despite our country predominantly using the WCV. Similar findings have been reported in other WCV-using countries, such as Colombia (34), Argentina (26), Russia (35), and Tunisia (36). Evidence from mouse models indicates that strains carrying *prn2*, in combination with *ptxP3*, exhibit enhanced colonization and greater fitness than *ptxP1/prn3* strains, regardless of the host’s immunization status (32). Interestingly, clinical isolates of *B. pertussis* with disrupted *prn* gene expression due to various independent mutations have been documented worldwide, including in countries such as the United States (37), Japan (38), China (39), Australia (27), Italy, France, Spain, and Belgium (40–42), where the ACV is administered. This phenomenon could be explained by findings from Hegerle et al., suggesting that not expressing pertactin provides *B. pertussis* with a selective advantage, potentially enhancing its early infection capabilities in hosts immunized with the ACV (43). Therefore, not only does the loss of Prn confer an advantage, but the specific *prn* type (*prn2*) also appears to enhance fitness in immunized mouse models (44), likely contributing to the success of *ptxP3/prn2* strains.

The *fim3* gene exhibits five allelic variants (*fim3-1*, *fim3-2*, *fim3-3*, *fim3-5*, and *fim3-6*), with *fim3-1* followed by *fim3-2*, the predominant variants globally (33, 45, 46), including in Latin American countries such as Colombia (34), where WCVs are part of the vaccination schedule. Consistent with these global trends, our study identified *fim3-1* as the predominant allele compared to *fim3-2* (97.3% vs 2.7%, respectively), reflecting the current use of WCVs in Peru (Fig. 2). This aligns with genomic studies showing *fim3-1* has historically predominated, while *fim3-2*, which emerged during the WCV period (~1% frequency), increased to 37% in countries adopting five-component ACVs. This contrasts with findings from Iran (47), which also uses WCVs, where *fim3-2* is predominant, as well as in Tunisia (36) and Argentina (26). Differences in the prevalence of *fim3* alleles, such as *fim3-2*, in countries using WCVs can be attributed to inter-country transmission, genetic divergence from vaccine strains, the immune status of the host population, selective pressure, or local strain adaptation to vaccine-induced immunity (33, 36, 48). For example, in countries like Australia and the United Kingdom, which primarily use three-component ACVs that do not include Fim2 and Fim3, *fim3-1* remains the predominant allele (48, 49), while the emergence of *fim3-2* has been linked to the transition from WCVs to five-component ACVs in the late 1980s (33). These findings suggest that the selective pressure exerted by vaccine composition may shape the genetic landscape of *B. pertussis*.

In this study, we identified two genotypes of *B. pertussis* circulating in Peru during 2018 and 2019: genotype VI (*ptxP3-ptxA1-prn2-fim3-1*), which predominated (96.8%), and genotype VII (*ptxP3-ptxA1-prn2-fim3-2*), which was present in relatively low proportions (Table 4; Fig. 3). These findings align with those of a previous study in Peru from 2012, which analyzed a smaller number of isolates (*n* = 18) and identified genotype VI predominantly in Ucayali (*n* = 5), Tacna (*n* = 3), Lima (*n* = 3), and Ayacucho (*n* = 1), whereas genotype VII was found in Ayacucho (*n* = 2), Piura (*n* = 2), Lima (*n* = 1), and Loreto (*n* = 1) (50). Our study, however, analyzed a larger sample size (*n* = 96) that was randomly selected from reported pertussis cases nationwide via a novel isolation-free typing method from direct clinical samples.

While our study revealed an increase in and predominance of genotype VI (96.8% from 2018 to 2019 compared with 66.7% in 2012), it also revealed a consistent genotypic profile with strains observed in 2012 (genotypes VI and VII). This consistency may be attributed to the highly monomorphic features of *B. pertussis* (33) and to its antigenic divergence, which likely arose under selective pressure induced by the introduction of the ACV (51). Interestingly, the predominance of genotype VI in Peru, where only the WCV is used, suggests that the expansion of *ptxP3*-carrying strains is not driven solely by selection pressure from the ACV (52). This observation is supported by the global presence and spread of genotype VI in countries employing either the WCV or ACV, including the United States (53, 54), Japan (55–57), China (28, 58), Argentina (26), Mexico (25), Colombia (34), Germany, Greece, Sweden, France, Spain, and Belgium (4, 5, 59–61). Furthermore, other *B. pertussis* genotypes, such as genotypes IV (*ptxP1-ptxA1-prn1-fim3-1*) and V (*ptxP1-ptxA1-prn3-fim3-1*), have been reported in some regions of China, where the ACV has been included in the immunization schedule since 2010 (28, 58, 62).

Although genotype VI differs from genotype VII by a single point mutation in *fim3*, this change has been associated with a clonal expansion, which coincided with a decrease in the diversity index from 0.66 to 0.35 (21). While we observed that a single genotype, focusing on vaccine-targeted loci, persisted over time, other non-vaccine-targeted genes could increase fitness and be responsible for the expansion. Genomic analysis of *B. pertussis* strains harboring the alleles *ptxA1*, *prn2/3*, and *ptxP3* revealed SNPs in non-vaccine-targeted genes, intergenic regions, and promoter regions, which could potentially affect both structure, regulation, and host-pathogen interactions (21, 63). Additionally, comprehensive genomic analysis across isolates from China, Finland, and the Netherlands identified gene loss over time, primarily located in protein-coding sequences associated with replication, recombination, repair, and transcription. The analysis also found the accumulation of over 100 SNPs in *ptxP3*-containing strains, mostly located in genes associated with transport and metabolism. These findings may explain the successful emergence of this lineage and its global spread (64).

While no statistically significant differences were found between genotypes VI and VII regarding age, genotype VI was more frequently observed in infants under 6 months (Table 5), a group known to experience more severe pertussis symptoms (2). This suggests that genotype VI may have biological characteristics that enhance infection, potentially linked to the single mutation in the *fim3* allele or other SNPs in vaccine non-targeted regions, as even minor mutations can significantly impact *B. pertussis* populations (21). Although no statistically significant differences were found in the proportions of genotypes VI and VII with respect to hospitalized and nonhospitalized infants, genotype VI was more frequently observed in hospitalized infants than in those who were not hospitalized (39 vs 12, respectively) (Table 5). However, these findings may reflect an uneven genotype distribution by age, geography, or other factors, rather than a direct relationship with disease severity. Further studies employing whole-genome sequencing and involving more representative and diverse populations are needed to clarify whether genotype VI may contribute to disease severity and to elucidate its distribution across various epidemiological contexts.

One limitation of this study is the nature of the sample. Stored nasopharyngeal swab samples may have degraded over time, potentially affecting the quantity and quality of DNA available for sequencing and thereby influencing the obtained results. Additionally, the samples analyzed may not fully represent the entire Peruvian population, introducing potential biases. However, this study utilized randomly selected samples (*n* = 96) from nationwide pertussis cases (*n* = 690) reported to the National Institute of Health for diagnostic confirmation between 2018 and 2019. These cases originated from most regions of Peru (23/25), including those with the highest pertussis caseloads, such as Lima, Loreto, and La Libertad. However, as these samples were predominantly collected from hospitals and healthcare facilities, they may not fully represent all *B. pertussis* infections occurring in the community. This limitation is a consequence of potential differences between genotypic variants present in individuals who do not seek medical care and those detected in patients attending healthcare facilities, potentially impacting the assessment of genotypic diversity.

Moreover, this study did not assess whether the circulating genotype in clinical samples exhibited Prn loss. While PCR-based sequencing can be labor-intensive, especially with large sample sizes, and less comprehensive than next-generation sequencing (NGS), it remains a cost-effective and practical alternative in resource-limited settings without NGS capabilities. Furthermore, isolating *B. pertussis* for NGS characterization remains challenging in such settings. Despite these constraints, PCR-based Sanger sequencing directly from clinical samples offers a feasible tool for monitoring genotypic shifts in circulating strains.

In conclusion, this study provides valuable insights into the genotypic variability of *B. pertussis* in Peru from 2018 to 2019, employing an isolation-free genotyping method based on the sequencing of vaccine antigen genes directly from clinical samples. This finding underscores the importance of ongoing and comprehensive genetic surveillance of *B. pertussis* in the country to guide public health strategies. Future research should prioritize expanding nationwide sample collection and analyzing longitudinal genotypic trends to enhance our understanding of the pathogen’s evolution dynamics in the region.

## Data Availability

All nucleotide sequences generated in this study have been deposited in the GenBank database under accession numbers PQ114763 to PQ115078.
